# In vitro investigation of relationship between quorum-sensing system genes, biofilm forming ability, and drug resistance in clinical isolates of *Pseudomonas aeruginosa*

**DOI:** 10.1186/s12866-024-03249-w

**Published:** 2024-03-25

**Authors:** Jaber Hemmati, Mohsen Nazari, Fatemeh Sadat Abolhasani, Amjad Ahmadi, Babak Asghari

**Affiliations:** 1 Student Research Committee, Hamadan University of Medical Sciences, Hamadan, Iran; 2 Department of Microbiology, School of Medicine, Hamadan University of Medical Sciences, Hamadan, Iran; 3 Department of Pathobiology, School of Public Health, Tehran University of Medical Sciences, Tehran, Iran

**Keywords:** *Pseudomonas aeruginosa*, Quorum sensing system, Biofilm formation, Drug resistance

## Abstract

**Background:**

*Pseudomonas aeruginosa* is an opportunistic pathogen in the health-care systems and one of the primary causative agents with high mortality in hospitalized patients, particularly immunocompromised. The limitation of effective antibiotic administration in multidrug-resistant and extensively drug-resistant *P. aeruginosa* isolates leads to the development of nosocomial infections and health problems. Quorum sensing system contributes to biofilm formation, expression of bacterial virulence factors, and development of drug resistance, causing prolonged patient infections. Therefore, due to the significance of the quorum sensing system in increasing the pathogenicity of *P. aeruginosa*, the primary objective of our study was to investigate the frequency of quorum sensing genes, as well as the biofilm formation and antibiotic resistance pattern among *P. aeruginosa* strains.

**Methods:**

A total of 120 *P. aeruginosa* isolates were collected from different clinical specimens. The disk diffusion method was applied to detect the antibiotic resistance pattern of *P. aeruginosa* strains. Also, the microtiter plate method was carried out to evaluate the biofilm-forming ability of isolates. Finally, the frequency of *rhlI*, *rhlR*, *lasI*, and *lasR* genes was examined by the polymerase chain reaction method.

**Results:**

In total, 88.3% *P. aeruginosa* isolates were found to be multidrug-resistant, of which 30.1% had extensively drug-resistant pattern. The highest and lowest resistance rates were found against ceftazidime (75.0%) and ciprofloxacin (46.6%), respectively. Also, 95.8% of isolates were able to produce biofilm, of which 42.5%, 33.3%, and 20.0% had strong, moderate, and weak biofilm patterns, respectively. The frequency of quorum sensing genes among all examined strains was as follows: *rhlI* (81.6%), *rhlR* (90.8%), *lasI* (89.1%), and *lasR* (78.3%). The most common type of quorum sensing genes among multidrug-resistant isolates were related to *rhlR* and *lasI* genes with 94.3%. Furthermore, *rhlI*, *rhlR*, and *lasI* genes were positive for all extensively drug-resistant isolates. However, the *lasR* gene had the lowest frequency among both multidrug-resistant (83.0%) and extensively drug-resistant (90.6%) isolates. Moreover, *rhlR* (94.7%) and *lasR* (81.7%) genes had the highest and lowest prevalence among biofilm-forming isolates, respectively.

**Conclusion:**

Our findings disclosed the significantly high prevalence of drug resistance among *P. aeruginosa* isolates. Also, the quorum sensing system had a significant correlation with biofilm formation and drug resistance, indicating the essential role of this system in the emergence of nosocomial infections caused by *P. aeruginosa*.

## Introduction

*Pseudomonas aeruginosa* (*P. aeruginosa*) is a primary common nosocomial pathogen that has become a big challenge for health**-**care systems due to causing death in immunocompromised, burn, and cystic fibrosis patients [1–3]. According to the Centers for Disease Control (CDC) reports, about 50,000 nosocomial *P. aeruginosa* infections occur annually in the United States [4]. The prevalence of multidrug-resistant or extensively drug-resistant *P. aeruginosa* (MDRPA/XDRPA) strains among hospitalized patients is a major public health concern [5]. These resistant strains are associated with significant morbidity and mortality, making it challenging to select appropriate treatments [6, 7]. The ability of biofilm formation, dissemination of transferable resistance determinants, and patient-to-patient transmission of resistant strains are among the main mechanisms for developing MDR and XDR patterns [8].

The adhesion of *P. aeruginosa* over different surfaces is due to the aggregation of the community of bacteria, which is described as biofilm [9, 10]. *P. aeruginosa* is a well-known biofilm producer, which efficiently adheres to biotic and abiotic surfaces and causes persistent and complicated infections [11]. Extracellular polymeric substance (EPS) is the structural characteristic of biofilm that shields embedded bacteria from the bactericidal or bacteriostatic activity of the antimicrobial agents [12]. In this regard, biofilm-associated *P. aeruginosa* infections are 20–30% more resistant to fluoroquinolones than those caused by planktonic cells [13]. Indeed, EPS provides a definite matrix structure with a barrier function and prevents antibiotic penetration [14]. *P. aeruginosa*, through cell-to-cell signaling, could form a bacterial community together for biofilm formation. This cell-density-dependent mechanism is known as quorum sensing (QS) that controls the expression of different genes [15].

*P. aeruginosa* employs the QS system as a vital regulatory mechanism for coordinating various cellular processes, including formation of biofilms, where the bacterial communities could significantly resist the antimicrobial agents and host immune responses. QS system involves producing and detecting of small signaling molecules known as autoinducers, which enable bacteria to communicate and synchronize their behaviors based on population density [16]. *Rhl* and *Las* are two major QS systems of *P. aeruginosa*, which have N-butanoyl-L-homoserine lactone (C4-HSL) and N-(3-oxododecanoyl)-L-homoserine lactone (3O-C12- HSL) as autoinducers, respectively [17]. These signaling molecules are separately synthesized by *RhlI* and *LasI*, and interact with their receptors (*RhlR* and *LasR*, respectively) when achieve the threshold concentration. The *Rhl* system participates in different stages of biofilm formation, including the production of microcolonies, maintenance of the open-channel architectures, and detachment of embedded bacterial cells. Also, *Rhl* controls *P. aeruginosa* virulence factors, such as pyocyanin, rhamnolipid, elastase, and hydrogen cyanide [18]. The *Las* system involves in other aspects of the biofilm formation process, such as biofilm maturation, and also regulates genes encoding exotoxin A, elastase, and alkaline protease [19]. Furthermore, QS mediates the regulation of drug efflux pumps and antibiotic-modifying enzymes, which could contribute to developing of drug resistance in *P. aeruginosa* [20].

The QS system regulates the expression of virulence factors, biofilm formation, and drug resistance mechanisms, which could present unique challenges in health-care systems. Also, drug resistance development limits the effectiveness of available antibiotics against *P. aeruginosa* infections, making treatment options more complicated [21]. Unraveling the interplay between the QS system, biofilm-forming ability, and drug resistance has the potential to revolutionize the management of *P. aeruginosa* infections, offering new avenues for combating the growing threats caused by this pathogen [22]. Therefore, considering the undeniable role of MDRPA and XDRPA strains in developing clinical challenges and the lack of effective treatment against infections caused by them, the present study aimed to evaluate the molecular identification of QS genes and their role in biofilm formation and drug resistance among *P. aeruginosa* clinical isolates.

## Material and methods

### Bacterial isolates

In this cross-sectional study from September 2021-August 2022, a total of 120 clinical isolates of *P. aeruginosa* were collected from patients admitted to the three major teaching hospitals (Sina, Besat, Shahid Beheshti) in Hamadan, west of Iran. The isolates were sent to the laboratory of the Department of Microbiology, Faculty of Medicine, Hamadan University of Medical Sciences and stored in Tryptic soy broth (TSB) (Merck, Germany) with 15% glycerol at -20 °C for further phenotypic and molecular tests.

### Bacterial identification using phenotypic and molecular tests

Laboratory identification of *P. aeruginosa* isolates was carried out using standard and routine bacteriological analyses. For this purpose, the collected isolates were first examined for colonial morphology, hemolysis, Gram staining, cytochrome oxidase and catalase reactions, growth on cetrimide agar, and pyocyanin pigment production [23]. Also, the biochemical reactions in microbial culture media, including triple sugar iron (TSI) agar, sulfide-indole-motility (SIM) agar, Kligler's iron agar (KIA), lysine iron agar (LIA), Simon citrate agar, and oxidative-fermentative (OF) were used to confirm isolates as *P. aeruginosa*. For final verification, the polymerase chain reaction (PCR) using specific primers for the *16S rRNA* gene of *P. aeruginosa* was also performed [24]. All *P. aeruginosa* isolates were stored in TSB medium with 15% glycerol at -20 °C for further investigations. Notably, all consumed culture media and reagents were purchased from Merck, Germany.

### Antimicrobial susceptibility testing

Antibiotic susceptibility profile was carried out for respective isolates by using the Kirby-Bauer disk diffusion method on Mueller–Hinton agar (Merck, Germany) as guidelines recommended by the Clinical and Laboratory Standards Institute (CLSI) [25]. *P. aeruginosa* ATCC 27853 was considered as a quality control strain, and six antimicrobial classes used in this study were as follows: cephalosporins (cefepime, ceftriaxone, cefoperazone, ceftazidime), aminoglycosides (amikacin, tobramycin, gentamycin), carbapenems (imipenem, ertapenem, meropenem), monobactams (aztreonam), fluoroquinolones (levofloxacin, ciprofloxacin, gatifloxacin, ofloxacin) and penicillins (piperacillin/tazobactam). Furthermore, the non-susceptible *P. aeruginosa* isolates to at least one agent in ≥ 3 and ≥ 6 different antimicrobial categories were defined as MDRPA and XDRPA, respectively [26, 27].

### Biofilm formation assay

The biofilm formation ability of collected isolates was investigated according to microtiter plate (MTP) assay [28]. Firstly, the isolates were inoculated into 5 ml TSB medium (Merck, Germany) supplemented with 1% glucose and incubated at 37 °C for 24 h. Afterward, the bacterial culture was diluted 1/100 with a sterile fresh TSB medium to prepare 10^6^ CFU/ml suspension. Subsequently, 200 µl of diluted suspension was distributed into a polystyrene 96-well microtiter plate (Jetbiofil, Canada), and after overnight incubation, the solution content of each well was discarded, and then, the wells were rinsed in triplicate with 200 μl sterile phosphate-buffered saline (PBS) (pH 7.4). Biofilms were fixed by absolute methanol (Merck Germany) for 15 min, and each well was stained with 200 μl of crystal violet solution (1.5%w/v) (Merck, Germany). After discarding the unbound stain, the stained biofilms were solubilized by adding 150 μl of acetic acid (33%v/v) (Merck, Germany) for 20 min. Finally, the optical densities (ODs) of each sample were determined using a microplate ELISA reader (Biotek, USA) at 620 nm and compared with the cut-off OD value (ODc). ODc was described as three standard deviations above the mean absorbance of the negative control, and uncollated TSB medium was used as a negative control. The biofilm formation pattern of isolates was classified into four groups according to the following formulas: OD < ODc (non-biofilm producer), ODc < OD < 2xODc (weak biofilm producer), 2xODc < OD < 4xODc (moderate biofilm producer), 4xODc < OD (strong biofilm producer). All assays were performed in triplicate, and *P. aeruginosa* ATCC 27853 was considered as a positive control.

### PCR for detection of QS genes

#### DNA extraction

DNA extraction was performed by using the boiling method [29]. Briefly, 1.5 ml of an overnight bacterial culture inoculated into a TSB medium was suspended in 500 μl of distilled water. Then, the bacterial cells were lysed by boiling for 10 min, and after centrifugation of all samples, the supernatants were stored at − 20 °C as DNA templates for amplification of studied genes.

#### PCR assay for quorum-sensing genes

The bacterial isolates were screened for QS genes, including (*rhlI*, *rhlR*, *lasI*, and *lasR*) by PCR assay. Four primer pairs were used to detect the investigated genes, and the specificity of each primer was confirmed using the NCBI Primer-BLAST [30]. The sequences of used primers and PCR programs for identifying different genes are presented in Tables 1 and 2. Finally, PCR products were analyzed by electrophoresis on 1% agarose gel (100v,1 h) supplemented with ethidium bromide (0.5 μg/ml), and allele size was estimated by a DNA ladder (100-1000 bp) (SinaClon, Iran). Notably, *P. aeruginosa* ATCC 27853 was used as a positive control for all examined genes.

**Table 1:** List of specific primers used in this study for detecting quorum-sensing genes

Genes	Primer sequence (5^′^ 3^'^)	Size of amplicon (bp)	Ref,
*rhlI*	F- TTCATCCTCCTTTAGTCTTCCC	155	[31]
	R- TTCCAGCGATTCAGAGAGC		
*rhlR*	F- TGCATTTTATCGATCAGGGC	133	[31]
	R- CACTTCCTTTTCCAGGACG		
*lasI*	F- CGTGCTCAAGTGTTCAAGG	295	[32]
	R- TACAGTCGGAAAAGCCCAG		
*lasR*	F- AAGTGGAAAATTGGAGTGGAG	130	[32]
	R- GTAGTTGCCGACGACGATGAAG

**Table 2:** PCR programs for detecting quorum-sensing genes in *P. aeruginosa* clinical isolates

Target gene	Initial denaturation (Temperature /time)	Denaturation (Temperature /time)	Annealing (Temperature /time)	Extension (Temperature /time)	Final extension (Temperature /time)	Cycle
*rhlI*	96 °C/3 min	96 °C/40 s	55 °C/1 min	72 °C/90 s	72 °C/10 min	35
*rhlR*	96 °C/3 min	96 °C/45 s	55 °C/55 s	72 °C/85 s	72 °C/10 min	35
*lasI*	94 °C/5 min	94 °C/1 min	56 °C/1 min	72 °C/1 min	72 °C/8 min	32
*lasR*	94 °C/5 min	95 °C/1 min	56 °C/50 s	72 °C/1 min	72 °C/8 min	32

### Statistical analysis

Statistical analysis was carried out using SPSS 16 software, and the rates of some parameters, such as MDR/XDR pattern, biofilm formation, and QS genes, were categorized. Also, the relationship between investigated parameters was assessed by Chi-squared and Fisher's exact tests. Notably, a statistically significant level was considered at *P*-value < 0.05 for all tests.

## Results

### Bacterial isolates

In total, 120 *P. aeruginosa* isolates were obtained from different medical wards (surgery, intensive care unit (ICU), burn, cardiovascular, infection disease, urology) and clinical sources, including blood, catheter, wound, CSF, urine, and trachea. The most of collected isolates were related to ICU ward, 44.1% (*n* = 53), and wound specimen, 27.5% (*n* = 33). Furthermore, the distribution of isolates by gender was 59.1% (*n* = 71) male and 40.8% (*n* = 49) female.

### Antimicrobial susceptibility pattern

Based on the disc diffusion test and CLSI guidelines, high levels of drug resistance were shown among our tested isolates. In this regard, 88.3% (106/120) *P. aeruginosa* isolates were detected as MDRPA, of which 30.1% (32/106) had XDRPA pattern. Ciprofloxacin, meropenem, and levofloxacin were the most effective antibiotics against examined isolates, with sensitivity rates as follows: 42.5%, 39.1%, and 31.6%. Also, the highest resistance was shown to ceftazidime, cefepime, and imipenem with the following rates: 75.0%, 72.5%, and 69.1%. The results of antibiotic resistance rates in *P. aeruginosa* clinical isolates are presented in Table 3.

**Table 3:** Contribution of 120 clinical isolates of *P. aeruginosa* to overall antimicrobial resistance rates

Classes/Antibiotics		Sensitive *N* (%)	Intermediate *N* (%)	Resistant *N* (%)
Cephalosporins	Cefepime	26 (21.6)	7 (5.8)	87 (72.5)
	Ceftriaxone	31 (25.8)	9 (7.5)	80 (66.6)
	Cefoperazone	29 (24.1)	10 (8.3)	81 (67.5)
	Ceftazidime	23 (19.1)	7 (5.8)	90 (75.0)
Aminoglycosides	Amikacin	28 (23.3)	10 (8.3)	82 (68.3)
	Tobramycin	31 (25.8)	8 (6.6)	81 (67.5)
	Gentamycin	29 (24.1)	12 (10.0)	79 (65.8)
Carbapenems	Imipenem	30 (25.0)	7 (5.8)	83 (69.1)
	Ertapenem	36 (30.0)	10 (8.3)	74 (61.6)
	Meropenem	47 (39.1)	13 (10.8)	60 (50.0)
Monobactams	Aztreonam	36 (30.0)	11 (9.1)	73 (60.8)
Fluoroquinolones	Levofloxacin	38 (31.6)	12 (10.0)	70 (58.3)
	Ciprofloxacin	51 (42.5)	13 (10.8)	56 (46.6)
	Gatifloxacin	35 (29.1)	11 (9.1)	74 (61.6)
	Ofloxacin	33 (27.5)	15 (12.5)	72 (60.0)
Penicillins	Piperacillin/tazobactam	32 (26.6)	9 (7.5)	79 (65.8)

### Correlation between biofilm formation with antibiotic resistance

**Fig. 1:**
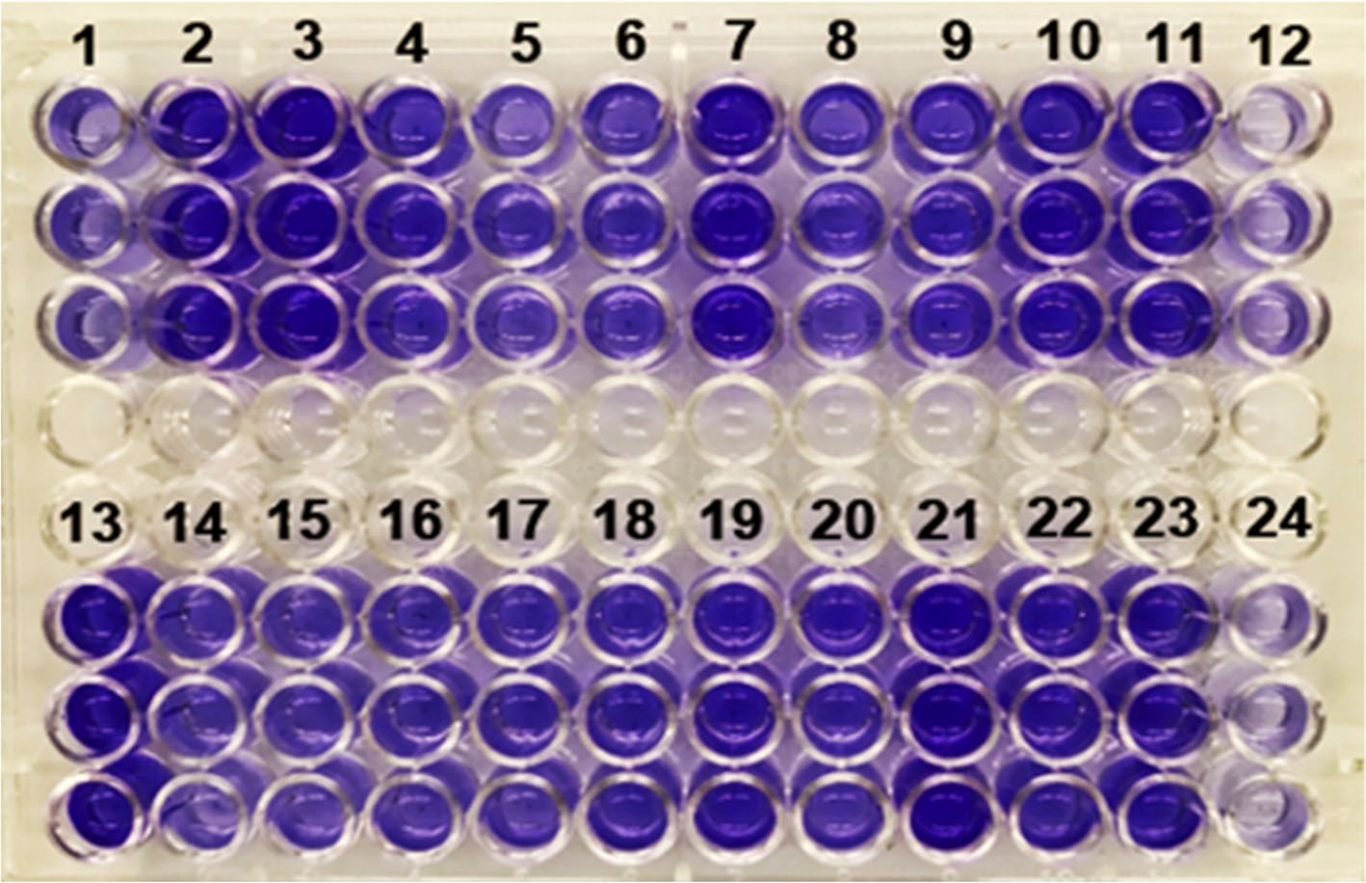
Results of microtiter plate (MTP) assay. Columns 1–10 and 13–22 represent the biofilm forming ability of tested isolates in triplicate. Columns 11 and 23 represent positive control (*P. aeruginosa* ATCC 27853). Columns 12 and 24 represent negative control (sterile tryptic soy broth medium)

According to MTP results (Fig. 1), the investigated clinical isolates had a high biofilm production potential, and the frequency of biofilm-forming isolates was 95.8% (*n* = 115). Also, 99.0% (*n* = 105) and 100% (*n* = 32) of MDRPA and XDRPA isolates were biofilm producers, respectively. As presented in Table 4, 46.2% of the biofilm-forming isolates with MDR patterns were strong biofilm producers, whereas 34.9% and 17.9% had moderate and weak biofilm patterns, respectively. In total, 90.6% and 9.3% of XDRPA isolates produced strong and moderate biofilm, respectively. The statistical analysis showed a significant relationship between biofilm formation and the MDR pattern (*P*-value < 0.001). However, there was no significant correlation between biofilm formation and XDRPA strains (*P*-value = 0.323).

**Table 4:** Correlation between biofilm formation ability with MDRPA and XDRPA clinical isolates

Biofilm Mode	MDRPA; No (%) (*n* = 106)	XDRPA; No (%) (*n* = 32)	Total (*n* = 120)
Strong	49 (46.2)	29 (90.6)	51 (42.5)
Moderate	37 (34.9)	3 (9.3)	40 (33.3)
Weak	19 (17.9)	0 (0)	24 (20.0)
Biofilm formation	105 (99.0)	32 (100)	115 (95.8)
*P*-value	< 0.001	0.323	**-**

### Correlation between QS genes with antibiotic resistance

**Fig. 2:**
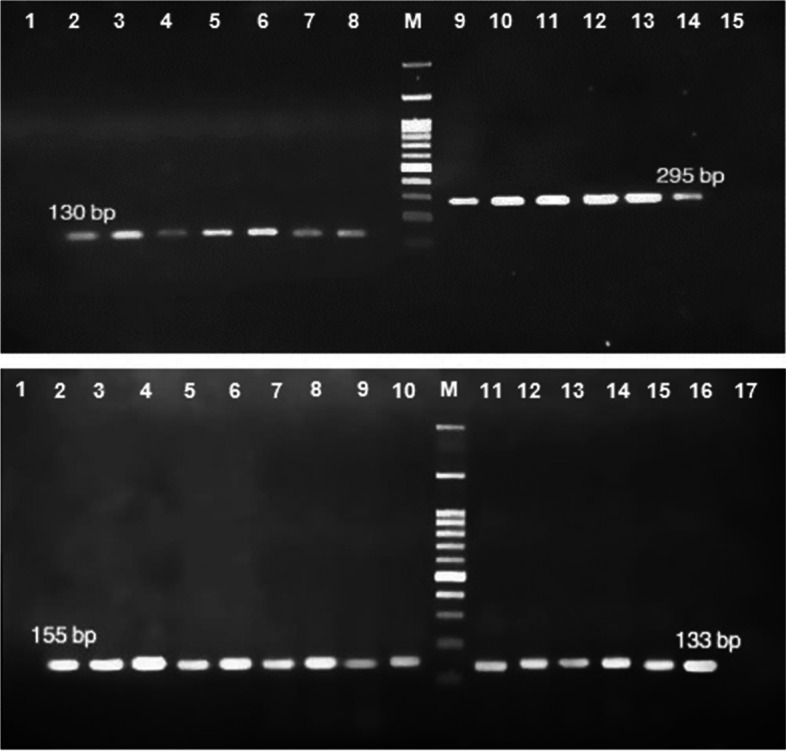
Polymerase chain reaction (PCR) assay for detection of quorum-sensing genes among *P. aeruginosa* isolates. Top: *lasR* with 130 bp and *lasI* with 295 bp; Lanes 2 and 14: PCR-positive control (*P. aeruginosa* ATCC 27853); Lanes 1 and 15: negative control; Lanes 3–8: positive strains with *lasR*; Lanes 9–13: positive strains with *lasI*. Down: *rhlI* with 155 bp and *rhlR* with 133 bp; Lanes 2 and 16: PCR-positive control (*P. aeruginosa* ATCC 27853); Lanes 1 and 17: negative control; Lanes 3–10: positive strains with *rhlI*; Lanes 11–15: positive strains with *rhlR*; Lane M: 100–1000 bp DNA ladder (SinaClon, Iran)

**Fig. 3:**
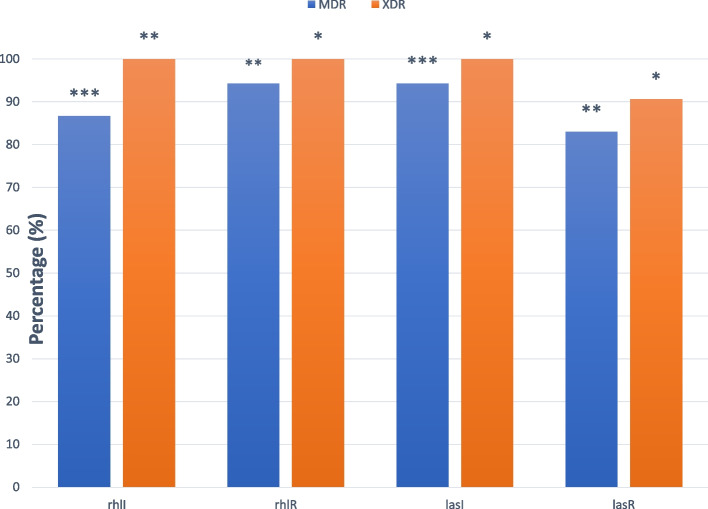
Statistically significant association between quorum-sensing genes distributions and MDRPA, XDRPA property (*P* value: * < 0.05, ** < 0.01, *** < 0.001)

In the present investigation, the prevalence of QS genes, including *rhlI*, *rhlR*, *lasI*, and *lasR*, was determined that the frequency of these genes among all clinical isolates of *P. aeruginosa* were 81.6%, 90.8%, 89.1%, and 78.3%, respectively. Figure 2 presents the PCR results and band patterns of QS genes among *P. aeruginosa* clinical isolates. Also, 70.8% (85/120) and 90.6% (29/32) of MDRPA and XDRPA isolates were positive for all QS genes, respectively. The highest frequency of investigated genes among MDRPA strains was related to *rhlR* and *lasI* genes with 94.3%*.* While*, lasR* gene, with 83.0%, has the lowest frequency among these isolates. On the other hand, all of the XDRPA isolates were positive for *rhlI*, *rhlR*, and *lasI* genes, but the frequency of *lasR* genes in these isolates was 90.6%. Furthermore, a significant correlation was found between the presence of QS genes and MDRPA strains (*P*-value = 0.013). In contrast, there was no significant relationship between QS genes and XDR property (*P*-value = 0.390). Figure 3 illustrates the frequency of *rhlI*, *rhlR*, *lasI*, and *lasR* among MDRPA and XDRPA isolates.

### Correlation between QS genes with biofilm formation

Our results revealed that 100% (*n* = 51) and 96.0% (*n* = 49) of *P. aeruginosa* isolates which were strong-biofilm producers, contained *rhlR* and *lasI* genes, respectively. However, the lowest prevalence of QS genes among strong biofilm-forming strains was related to *lasR* (78.4%), which had the highest prevalence among weak-biofilm producers (95.8%). Moreover, the highest presence of suited genes between moderate biofilm producers was related to *rhlI* (97.5%) and *rhlR* (95.0%), respectively. Overall, by comparing the frequency of QS genes among biofilm producer and non-biofilm producer isolates, our study proved the significant correlation between the presence of these genes (on overage = 88.4%) and biofilm formation (*P*-value = 0.008). Table 5 shows the comparative frequency of QS genes among biofilm producer *P. aeruginosa* clinical isolates.

**Table 5:** Frequency distribution of quorum-sensing genes among biofilm-forming *P. aeruginosa* isolates

Quorum -sensing genes	Biofilm mod			
	**Weak; No (%)** **(*n* = 24)**	**Moderate; No (%)** **(*n* = 40)**	**Strong; No (%)** **(*n* = 51)**	**Biofilm formation (%)** **(*n* = 115)**
***rhlI***	15 (62.5)	39 (97.5)	43 (84.3)	97 (84.3)
***rhlR***	20 (83.3)	38 (95.0)	51 (100.0)	109 (94.7)
***lasI***	21 (87.5)	37 (92.5)	49 (96.0)	107 (93.0)
***lasR***	23 (95.8)	31 (77.5)	40 (78.4)	94 (81.7)
***P*-value**	0.799	0.719	0.136	0.008

## Discussion

*P. aeruginosa* is an important drug-resistant bacterial pathogen, becoming a prominent life-threatening challenge for hospitalized patients. Infection with *P. aeruginosa* strains is a significant health concern for patients with immune-compromised status, cystic fibrosis, and burn injuries. This organism can persist in medical settings owing to its strong resistance to various antimicrobial agents, causing nosocomial infections with high mortality and morbidity rates [1, 2]. *P. aeruginosa*-related infections are usually complicated to treat due to high level of drug resistance, making it challenging to prescribe appropriate antibiotics [6]. As presented in this study, although all *P. aeruginosa* clinical strains showed high resistance rates to all tested antimicrobial classes (more than 50%), ciprofloxacin, meropenem, and levofloxacin were among the most effective antibiotics against these isolates. Also, the high resistance rates (more than 65.0%) were shown against aminoglycosides (amikacin, tobramycin, gentamycin), cephalosporins (cefepime, ceftriaxone, cefoperazone, ceftazidime), penicillins (piperacillin/tazobactam), and carbapenems (imipenem). Other reports from Iran were in agreement with the current study, which revealed *P. aeruginosa* isolates were resistant to a wide range of antimicrobial agents in our region [33, 34]. However, other studies showed different antibiotic resistance patterns among *P. aeruginosa* isolated in various parts of Iran [35, 36]. In this regard, the reports of a study in the north of Iran [37] showed the percentage of resistance to tested antibiotics as follows: amikacin 48.8%, tobramycin 58.2%, gentamicin 37.2%, ceftazidime 68.6%, and imipenem 23.3%. Moreover, our strains were more resistant than others isolated from different regions of the world and a study in Poland [38] showed the following resistance rates in *P. aeruginosa* clinical isolates; amikacin (30.7%), ceftazidime (33.2%), gentamycin (37.6%), tobramycin (38.1%), cefepime (42.6%), and piperacillin/tazobactam (39.6%). These contradictory results might be due to the absence of a comprehensive monitoring program for the appropriate antibiotic administration, differences in antibiotic prescription patterns, diversity of the geographical regions, and variety in methods used for measuring antibiotic susceptibility.

Fluoroquinolones are among the most efficient antibacterial compounds against various clinical bacterial pathogens, including *P. aeruginosa*. However, widespread administration of this antimicrobial group had an effective role in the outbreak of nosocomial infections caused by *P. aeruginosa* strains in recent years [39]. Modification of target enzymes, reduction in outer membrane permeability, and expressing efflux systems are known as the primary resistance mechanisms to fluoroquinolones in *P. aeruginosa* [40]. In the current study, the susceptibility to fluoroquinolones (on average, 32.6%) was higher than other classes of antibiotics. Besides, the resistance rates against four tested fluoroquinolones, including ciprofloxacin, levofloxacin, gatifloxacin, and ofloxacin, varied from 46.6% to 61.6%. Our antimicrobial susceptibility results showed that the most effective fluoroquinolone was ciprofloxacin, while in another study from Iran [41], the highest susceptibility rate among *P. aeruginosa* clinical isolates was related to ofloxacin. In agreement with our results, Rajaei et al. [42] in Iran reported that ciprofloxacin was the most effective agent against *P. aeruginosa* isolates. However, in a contradictory study in Palestine [43], all clinical *P. aeruginosa* isolates were resistant to ciprofloxacin and norfloxacin. These discrepancies indicate a difference in fluoroquinolones resistance patterns worldwide, so augmenting local antimicrobial stewardship and CLSI guidelines necessitates preventing the emergence of antimicrobial resistance among *P. aeruginosa* isolates.

The spread of MDRPA and XDRPA isolates in hospital environments has growing rates worldwide, usually causing prolonged hospitalization and invasive therapeutic procedures in infected patients. *P. aeruginosa* can employ several inherent and adaptive resistance mechanisms, allowing its survival under unfavorable conditions, such as host immune responses and antibiotic actions. The limited permeability of outer membrane, efflux pump systems, production of antibiotic-inactivating enzymes, and biofilm formation are among the resistance mechanisms in *P. aeruginosa*, leading to the emergence of MDRPA and XDRPA strains [44]. Another notable result of this research was the high incidence of drug resistance among all *P. aeruginosa* isolates. In this regard, 88.3% *P. aeruginosa* isolates were detected as MDRPA, of which 30.1% had XDRPA pattern. The MDRPA rate in current study was more than previous reports from Italy [45], Egypt [46], Canada [47], and China [48]. Also, our MDRPA rate was confirmed by other conducted studies in Iran [49, 50]. However, the XDRPA rate in this study was greater than the reports by Mirzaei et al. (15.5%) from Iran [51], Saleem et al. (18.1%) from Pakistan [52], and Royo-Cebrecos et al. (17.2%) from Spain [53]. These findings show the high prevalence of MDRPA/XDRPA isolates in our country and emphasize the urgent need for strong microbiological surveillance procedures, correct antibiotic prescription, and strict implementation of antimicrobial policies in health-care systems.

Biofilm formation is known as a major virulence factor in *P. aeruginosa*, which could significantly participate in bacterial pathogenicity. It has been proven that bacteria in biofilm form were 100–1000 fold more resistant than planktonic cells [54]. The bacterial cells grown in biofilm are able to tolerate the antimicrobial agents attack, which could lead to emergence of recurrent and latent infections in healthcare system. Notably, biofilm formation is one of the prominent adaptive resistance mechanisms in *P. aeruginosa*, resulting in numerous therapeutic obstacles related to this superbug [55]. In the present research, 99.0% (105/106) of MDRPA isolates were detected as biofilm producers, indicating a significant relationship between biofilm formation and MDRPA pattern (*P*-value < 0.001). Our results were consistent with Perez et al. [56] and Abidi et al. [57] studies, where the biofilm formation was significantly higher in MDRAP isolates. In contrast, in another published study, there was not a significant relationship between adhesion profile and MDRPA isolates, and other mechanisms, such as altered outer membrane permeability, efflux pumps, and toxin systems, were known as effective factors in developing drug resistance [31]. Overall, biofilm formation is among the reasons for the recurrence of *P. aeruginosa* infections that could create significant therapeutic challenges in hospitalized patients [58].

QS is a bacterial mechanism allowing to regulate gene expression based on cell-density population. *P. aeruginosa* possesses two main QS systems, *lasI-lasR* and *rhlI-rhlR*, which contribute to various cellular processes. Also, QS system has an important role in increasing bacterial virulence, leading to the limitation of therapeutic options against *P. aeruginosa* infections [44]. In our study, the frequency of QS system genes was as follows: *rhlI* (81.6%), *rhlR* (90.8%), *lasI*, (89.1%), and *lasR* (78.3%). In another study in India, the occurrence of QS genes among *P. aeruginosa* isolates was 41.6% for *rhlI* gene, 58.3% for *rhlR* gene, and 75% for *lasI* and *lasR* genes. In addition, similar results were shown in Egypt [59], where the prevalence of these genes was as follows: *rhlI* (40.0%), *rhlR* (36.0%), *lasI* (48.0%), and *lasR* (40.0%). Furthermore, our results disclosed that 65.8% (79/120) of tested isolates presented all the QS genes. By contrast, the findings reported by Perez et al. [60] showed that 90.1% (82/91) of the analyzed isolates had all the QS genes. Similarly, in the study by Lima et al. [31] in Brazil, there was a high occurrence of QS genes, and 97.5% (39/40) of isolates were positive for *rhlI*, *rhlR*, *lasI*, and *lasR*. The varied range in prevalence of these genes revealed that *P. aeruginosa* carries several mutations in the QS system.

QS system has an effective role in expression of different virulence factors in Gram-negative pathogens, including *P. aeruginosa.* This regulatory effect of QS system can be considered a resistance mechanism in *P. aeruginosa*, contributing to the emergence of drug-resistant isolates [61]. A prominent observation in the present study was a strong association between the presence of QS genes and antibiotic resistance pattern, where the genotypic analysis revealed that 70.8% of MDRPA isolates had all QS genes. In this regard, previously published studies [62, 63] demonstrated the essential role of the QS system in drug resistance development of *P. aeruginosa* through increasing bacterial pathogenicity, biofilm establishment, secretion of virulence factors, and regulating swarming motility. However, another study [64] showed that QS-deficient strains tended to be less susceptible to antimicrobial agents. In addition, in our study, despite 90.6% of XDRPA strains were positive for all investigated genes, there was no correlation between XDR pattern and presence of QS genes. This contradiction is possibly attributed to this fact that other mechanisms including expression of antimicrobial resistance genes, restriction of outer-membrane permeability, and release of antibiotic-inactivating enzymes may be involved in development of resistant strains.

*P. aeruginosa* strains have a high ability to form biofilm on various surfaces, which can increase the mortality risk among hospitalized patients. Regulation of biofilm formation in *P. aeruginosa* mainly depends on the QS system, which could be involved in the initial cell–cell adhesion. Also, the *las-rhl* system is activated during the irreversible attachment and contributes to biofilm maturation [44]. In the current study, we observed a significant correlation between the presence of QS genes and biofilm formation ability of *P. aeruginosa* isolates. Likewise, the genotypic analysis revealed that the prevalence of four QS genes among biofilm producers was as follows: *rhlI* (84.3%), *rhlR* (94.7%), *lasI* (93.0%), and *lasR* (81.7%). Based on previously published studies [60, 65], the rates of these genes among biofilm-forming isolates were high, and the correlation between the QS gene and biofilm production was approved. In this regard, Lima et al. [31] indicated that 100% of biofilm-forming strains were positive for *rhlI*, *rhlR*, and *lasR* genes, and the *lasI* gene was detected in 97.5% of them. In addition, another study [66] showed that 100% of biofilm-forming *P. aeruginosa* strains possessed *rhlI*, *rhlR*, *lasI*, and *lasR* genes. Also, Alayande et al. [67] found a strong relationship between QS signal molecules (3OC12-HSL, C6-HSL) and biofilm development. However, Karami et al. [35] demonstrated that biofilm production was irrelevant for QS genes. In agreement with this finding, the studies indicated that *P. aeruginosa lasR*/*lasI*-mutant were biofilm producers [31, 60]. Also, Fattouh et al. detected two biofilm-producer *P. aeruginosa* strains that were negative for all QS genes [68]. These contradictions indicate that other QS systems, such as the *Pseudomonas* quinolone signal (PQS), as well as other genes in the *las*/*rhl* operon could play a role in *P. aeruginosa* biofilm formation.

Based on our findings, future research could focus on elucidating the precise molecular mechanisms underlying the interactions between signaling molecules, receptors, and transcription factors involved in QS gene regulation. Furthermore, understanding the environmental factors that influence the QS system could open new avenues for developing targeted strategies to manipulate bacterial behavior and disrupt QS-mediated processes, such as biofilm formation, emerging drug resistance pattern, and virulence factor production. Despite the valuable insights gained from this study on the significant regulatory mechanism of QS genes in *P. aeruginosa* pathogenesis, it is important to acknowledge several limitations that may impact the interpretation of our findings. Firstly, the sample size used in this study could limit the generalizability of the results to larger patients' population. Additionally, there may have been inherent biases in the sample selection process, potentially affecting the representation of certain bacterial isolates. Moreover, this study focused on a specific geographical region for patients, which may restrict the applicability of our findings to the broader contexts. Also, the determination of expression levels of QS genes by quantitative real-time PCR can help to clarify the exact regulatory mechanisms of the QS system in biofilm formation and drug resistance development, which our study only examined the prevalence of these genes.

## Conclusion

In conclusion, our investigation into the epidemiology of antibiotic susceptibility patterns, biofilm formation, and QS genes among clinical *P. aeruginosa* isolates in Hamadan, west of Iran, unveiled a significantly high prevalence of MDRPA and XDRPA strains. Furthermore, our study revealed a substantial occurrence of *P. aeruginosa* biofilm producers among examined isolates. Notably, we observed a prominent correlation between the QS system with biofilm formation and drug resistance in *P. aeruginosa*, underscoring the critical role of this system in increasing therapeutic challenges related to MDRPA and XDRPA strains. However, future studies could aim to overcome our study limitations by employing a larger and more diverse sample size, exploring a wide-range of environmental conditions, and determining the expression levels of QS genes. By addressing these limitations, future studies can provide a more comprehensive understanding of the regulatory mechanisms of QS genes and their implications in treating *P. aeruginosa* infections. Finally, it is imperative to discover efficient strategies to disrupt QS-mediated bacterial pathogenesis, leading to the development of novel therapeutic approaches aimed at combating nosocomial infections caused by *P. aeruginosa*.

## Data Availability

All data generated or analyzed during this study were included in this article but the raw data are available from the corresponding author on reasonable request.
